# Factors affecting nursing and health technician students' satisfaction with distance learning during the COVID-19 pandemic in Morocco: a descriptive study

**DOI:** 10.3352/jeehp.2022.19.28

**Published:** 2022-10-17

**Authors:** Aziz Naciri, Mohamed Radid, Abderrahmane Achbani, Mohamed Amine Baba, Ahmed Kharbach, Ghizlane Chemsi

**Affiliations:** 1 Laboratory of Sciences and Technologies of Information and Education, Faculty of Sciences Ben M’Sik, Hassan II University of Casablanca, Casablanca, Morocco; 2 Laboratory of Physical Chemistry of Materials, Faculty of Sciences Ben M’Sik, Hassan II University of Casablanca, Casablanca, Morocco; 3 Laboratory of Cell Biology and Molecular Genetics, Department of Biology, Faculty of Sciences, Ibn Zohr University, Agadir, Morocco; 4 Laboratory of Health Sciences Research, Faculty of Medicine and Pharmacy of Agadir, Ibn Zohr University, Agadir, Morocco; 5 Laboratory of Biostatistics, Clinical Research and Epidemiology, Faculty of Medicine and Pharmacy of Rabat, Mohamed V University, Rabat, Morocco; Hallym University, Korea

**Keywords:** Computer-assisted instruction, COVID-19, Morocco, Personal satisfaction, Nursing students

## Abstract

**Purpose:**

Distance learning describes any learning based on the use of new multimedia technologies and the internet to allow students to acquire new knowledge and skills at a distance. This study aimed to determine satisfaction levels with distance learning and associated factors among nursing and health technician students during the coronavirus disease 2019 pandemic in Morocco.

**Methods:**

An descriptive study was conducted between April and June 2022 among nursing and health technician students using a self-administered instrument. The student satisfaction questionnaire consists of 24 questions categorized into 6 subscales: instructor, technology, course setup, interaction, outcomes, and overall satisfaction. It was based on a 5-point Likert scale, ranging from 1 (strongly disagree) to 5 (strongly agree). Univariate and multivariate logistic regression analyses were conducted to identify factors associated with student satisfaction during distance learning.

**Results:**

A total of 330 students participated in this study, and 176 students (53.3%) were satisfied with the distance learning activities. A mean score higher than 2.8 out of 5 was obtained for all subscales. Multiple regression analysis showed that students’ year of study (adjusted odds ratio [aOR], 2.34; 95% confidence interval [CI], 1.28–4.27) and internet quality (aOR, 0.47; 95% CI, 0.29–0.77) were the significant factors associated with students’ satisfaction during distance learning.

**Conclusion:**

This study highlights the satisfaction level of students and factors that influenced it during distance learning. A thorough understanding of student satisfaction with digital environments will contribute to the successful implementation of distance learning devices in nursing.

## Introduction

### Background/rationale

The mass implementation of distance learning activities in nursing education institutions during the coronavirus disease 2019 (COVID-19) pandemic requires specific intent. Indeed, student satisfaction with distance learning is a critical component of student engagement and success [1,2]. Previous research findings before the pandemic were divergent, and some studies showed that students were satisfied both with distance learning and in face-to-face courses [3,4]. Other investigations reported that students are more satisfied with a face-to-face course than with an online one [5,6]. A systematic review showed that health students gave positive opinions on online learning during COVID-19 regarding perspectives, acceptability, motivation, and involvement [7]. Others have reported that factors such as interactions in the online class, student motivation to participate in the online class, course structure, and instructor facilitation and knowledge were related to student satisfaction [8].

### Objectives

The study aimed to examine students’ satisfaction levels with distance learning and identify factors associated with students’ satisfaction with distance learning during the COVID-19 pandemic in Morocco.

## Methods

### Ethics statement

This study was approved by the Higr Institute of Nursing Professions and Technical Health in Laayoune (25-Apr-22). Informed consent was obtained from all participants.

### Study design

An descriptive, quantitative, single-center study was conducted using a self-administered questionnaire. The study was described according to the STROBE (Strengthening the Reporting of Observational Studies in Epidemiology) statement [9].

### Setting

This study was conducted among students at the High Institute of Nursing Professions and Technical Health in Laayoune, Morocco. Data were collected from April 26, 2022, to May 30, 2022 (Dataset 1).

### Participants

This research included all students attending distance learning courses at the High Institute of Nursing Professions and Technical Health in Laayoune, Morocco for the academic year 2021/2022. There was no exclusion criterion.

### Variables

The 24 items of the questionnaire were classified into the following subscales: instructor, technology, course setup, interaction, outcomes, and overall satisfaction.

### Data sources/measurement

The measurement instrument used is a self-administered questionnaire divided into 2 parts (Supplement 1). The first part contained items on participants’ socio-demographic and learning experience data such as age, gender, nationality, discipline, specialty, year of study, the educational platform used, and internet quality. The second part contained items from the students’ satisfaction questionnaire developed by Bolliger and Halupa [10]. It consisted of 24 items categorized into the following subscales: instructor, technology, course setup, interaction, outcomes, and overall satisfaction. The questionnaire used a 5-point Likert scale, ranging from 1 (strongly disagree) to 5 (strongly agree). The student satisfaction questionnaire’s reliability was high (Cronbach α=0.91), and the reliability of all subscales was acceptable: instructor (α=0.82), technology (α=0.76), course setup (α=0.60), interaction (α=0.60), outcomes (α=0.72), and overall satisfaction (α=0.85) [10].

### Bias

No selection bias was identified. The study included all students who agreed to participate and satisfied the study’s eligibility criteria.

### Study size

Since all target participants were invited and all of them re-sponded, sample size estimation was not estimated.

### Statistical methods

The qualitative variables were presented as frequency, percentages, and mean±standard deviation or median (interquartile range, IQR) for quantitative variables. The chi-square test was performed to identify differences in proportions of categorical variables between 2 groups (satisfied, not satisfied). This classification was conducted using the dynamic clustering method after calculating the total satisfaction score for each participant. Moreover, univariate and multivariate logistic regression analyses were carried out to identify the factors associated with student satisfaction with distance learning. The multivariate logistic regression analysis considered all independent variables with a P-value less than 0.25 in the univariate analysis. P-values less than 0.05 were considered to indicate statistical significance. Data management and statistical analysis were conducted using SPSS ver. 13.0 (SPSS Inc., Chicago, IL, USA).

## Results

### Participants

Of 330 participants in this study, 249 (75.5%) were female, with a mean age of 20.2±1.3 years (Table 1). Most of the students 323 (97.9%) were Moroccan. Students from all nursing and health technician specialties participated in the study: 189 (57.3%) were training to become generalist nurses, 60 (18.2%) to become nurses in anesthesia and intensive care, 47 (14.2%) to become nurses in emergency and intensive care, 19 (58%) to become midwives, 8 (2.4%) to become radiology technicians, and 7 (2.1%) to become laboratory technicians. Regarding the level of study, 141 (42.7%) of the students were in the 1st year, 97 (29.4%) in the 2nd year, and 92 (27.9%) in the 3rd year.

### Main results

Of the 330 students, 176 (53.3%) were satisfied with the distance learning activities, of whom 128 (51.4%) were female. Generalist nursing students represented 57.3% of the sample, of which 58.7% were satisfied with distance learning. More than half (60.3%) reported using the WhatsApp application, with which 53.3% were satisfied. Furthermore, 33.6% of the participants combined several educational platforms, and 55% of them were satisfied with this method. Concerning internet quality, 115 out of 192 (59.9%) participants declared that the internet connection was good and expressed their satisfaction with distance learning. However, statistically significant differences were detected according to the student’s year of study (P=0.016) and the internet quality (p=0.008) (Table 1). Table 2 presents the student satisfaction subscales. Overall satisfaction was the subscale with the highest mean score (2.98±0.75) with a median of 3 (IQR, 2.5–3.5), followed by the outcomes subscale (2.94±0.80) with a median of 3 (IQR, 2.5–3.5). A mean score greater than 2.8 out of 5 was reported in all subscales.

The results of the univariate regression analysis presented in Table 3 show a possible association between: age (odds ratio [OR], 1.55; 95% confidence interval [CI], 0.83–2.91), gender (OR, 1.38; 95% CI, 0.83–2.29), year of study (OR, 1.57; 95% CI, 0.92–2.70), internet quality (OR, 0.56; 95% CI, 0.23–1.34) and the student’s satisfaction during distance learning. The multiple regression analysis showed that the student’s year of study (2nd year: adjusted odds ratio [aOR], 2.34; 95% CI, 1.28–4.27) and internet quality (good: aOR, 0.47; 95% CI, 0.29–0.77) were the significant factors associated with the students’ satisfaction during distance learning (Table 4). The coefficient determinant of logistic regression (Nagelkerke R^2^) was 0.083, and the precision of prediction in this study was 59.7%.

## Discussion

### Key results

The present study aimed to determine students’ level of satisfaction with distance learning and the factors associated with this satisfaction. The multivariate logistic regression analysis showed that the students’ year of study and internet quality were significant predictors of student satisfaction. The mean scores of all subscales were above 2.8 out of 5 for all components. More than half of the participants (53.3%) were satisfied with the distance learning activities.

### Interpretation

We concluded that nursing and health technician students were satisfied with distance learning. This result may be due to the perceived safety of the students during distance learning compared to face-to-face learning during the COVID-19 period. However, the communication and flexibility offered by distance learning can also potentially influence perceptions of satisfaction among students. The study also concluded that the year of study was associated with student satisfaction. This may be due to the progress through the program. In other words, using information technology tools at a distance becomes habitual for students in the 2nd year. Regarding internet quality, which was also associated with satisfaction, a good internet connection may facilitate the visualization and downloading of pedagogical materials (audio, videos, PPT files, PDF files) provided by the instructors.

### Comparison with previous studies

The study conducted by Amir et al. reported, on the one hand, that students were more satisfied with traditional classroom teaching than with distance learning and that first-year students are more likely to prefer distance learning. These revelations are in contrast to our results. On the other hand, it also concluded that internet connection quality was among the factors that caused challenges in distance learning, which is consistent with the findings of our study [7]. Moreover, another study found that student-teacher interaction, teacher performance, course evaluation, design, and technique were factors influencing student satisfaction [11]. In our study, students showed satisfaction regarding the interactions, instructors, course design, and technology. Another investigation stated that internet access is related to student satisfaction [12]. Indeed, this finding is in accordance with our results, showing that internet quality is a factor associated with student satisfaction with online teaching activities.

### Limitations

This study had some limitations. First, the distance courses were rapidly implemented in response to needs without prior preparation by instructors or students. Second, the study did not consider the designs of the courses taught at a distance (synchronous/asynchronous). Third, the study focused on distance learning of declarative, procedural, and conditional knowledge sets. These limitations may influence our results, which should be interpreted with caution.

### Generalizability

Although this was a single-center study, the results may be able to be generalized to all Moroccan nursing and health technician students if they participate in distance learning.

### Suggestions

Distance learning is a required educational method in health professionals’ education. Further similar research is recommended to study satisfaction with synchronous and asynchronous distance learning. Furthermore, reflections on how to develop the technical skills of distance students are highly recommended.

### Conclusion

Distance learning was the alternative to face-to-face teaching during the COVID-19 health crisis. Many students were experiencing this for the first time. The study found that nursing and health technician students were satisfied with distance learning during the COVID-19 pandemic in Morocco despite the conditions of its introduction. The year of study and the internet quality were the factors associated with their satisfaction.

## Figures and Tables

**Table 1.** Participants’ characteristics based on their satisfaction with distance learning

Characteristic	No. (%)	Satisfied no. (%)	Not satisfied no. (%)	P-value
Age (yr, mean±standard deviation)	20.2±1.3			
Age group (yr)				0.168
<21		146 (51.8)	136 (48.2)	
>21		30 (62.5)	18 (37.5)	
Gender				0.128
Female	249 (75.5)	128 (51.4)	121 (48.6)	
Male	81 (24.5)	48 (59.3)	33 (40.7)	
Nationality				0.332
Moroccan	323 (97.9)	171 (52.9)	152 (47.1)	
Other	7 (2.1)	5 (71.4)	2 (28.6)	
Specialty				0.082
Generalist nurse	189 (57.3)	111 (58.7)	78 (41.3)	
Midwife	19 (5.8)	6 (31.6)	13 (68.4)	
Nurse in anesthesia and intensive care	60 (18.2)	25 (41.7)	35 (58.3)	
Emergency and critical care nurse	47 (14.2)	26 (55.3)	21 (44.7)	
Radiology technician	8 (2.4)	5 (62.5)	3 (37.5)	
Laboratory technician	7 (2.1)	3 (42.9)	4 (57.1)	
Year of study				0.016
1st year	141 (42.7)	75 (53.2)	66 (46.8)	
2nd year	97 (29.4)	42 (43.3)	55 (56.7)	
3rd year	92 (27.9)	59 (64.1)	33 (35.9)	
Platform or application used				0.806
WhatsApp	199 (60.3)	106 (53.3)	93 (46.7)	
Zoom cloud meeting	10 (3.0)	5 (50.0)	5 (50.0)	
Moodle	1 (0.3)	0	1 (100.0)	
Google Meet	3 (0.9)	1 (33.3)	2 (66.7)	
Edmodo	5 (1.5)	2 (40.0)	3(60.0)	
Google Classroom	1 (0.3)	1 (100.0)	0	
Combining several platforms	111 (33.6)	61 (55.0)	50 (45.0)	
Internet quality				0.008
Excellent	25 (7.6)	14 (56.0)	11 (44.0)	
Good	192 (58.2)	115 (59.9)	77 (40.1)	
Poor	113 (34.2)	47 (41.6)	66 (58.4)	

**Table 2.** Subscales of student satisfaction

Subscales	Mean±standard deviation	Median (interquartile range)
Instructor	2.88±0.62	2.75 (2.5–3.25)
Technology	2.9±0.68	3.0 (2.5–3.25)
Course setup	2.82±0.67	2.75 (2.25–3.25)
Interaction	2.85±0.67	2.75 (2.5–3.25)
Outcomes	2.94±0.80	3.0 (2.5–3.5)
Overall	2.98±0.75	3.0 (2.5–3.5)

**Table 3.** Satisfaction and associated factors in univariate logistic regression analysis

Variable	Odds ratio (95% confidence interval)	P-value
Age (yr)		
<21	1.55 (0.83–2.91)	0.171
>21	1	
Gender		
Female	1.38 (0.83–2.29)	0.219
Male	1	
Nationality		
Moroccan	2.22 (0.42–11.62)	0.344
Other	1	
Specialty		
Generalist nurse	0.53 (0.11–2.42)	0.410
Midwife	1.62 (0.27–9.66)	0.593
Nurse in anesthesia and intensive care	1.05 (0.22–5.11)	0.952
Emergency and critical care nurse	0.61 (0.12–3.01)	0.540
Radiology technician	0.45 (0.06–3.57)	0.450
Laboratory technician	1	
Year of study		
1st year	1.57 (0.92–2.70)	0.100
2nd year	2.34 (1.30–4.2)	0.004
3rd year	1	
Platform or application used		
WhatsApp	1.07 (0.67–1.71)	0.775
Zoom cloud meeting	1.22 (0.33–4.45)	0.763
Moodle		
Google Meet	2.44 (0.21–27.7)	0.472
Edmodo	1.83 (0.29–11.38)	0.517
Google Classroom	0.00 (0.00–0.00)	1.000
Combining several platforms	1	
Internet quality		
Excellent	0.56 (0.23–1.34)	0.193
Good	0.48 (0.3–0.76)	0.002
Poor	1	

**Table 4.** Factors associated with students’ satisfaction with distance learning using multivariate logistic regression analysis

Variable	Adjusted odds ratio (95% confidence interval)	P-value
Year of study: 2nd year	2.34 (1.28–4.27)	0.006
Internet quality: good	0.47 (0.29–0.77)	0.003

## Footnotes

**Data availability**
Data files are available from Harvard Dataverse: https://doi.org/10.7910/DVN/GVZJSH Dataset 1. Students’ response to a questionnaire on satisfaction with distance learning during the COVID-19 pandemic in Morocco.

## Supplementary materials

Supplementary files are available from Harvard Dataverse: https://doi.org/10.7910/DVN/GVZJSH

Supplement 1. Survey questionnaires on satisfaction with distance learning during the COVID-19 pandemic in Morocco.

Supplement 2. Audio recording of the abstract.
