# Supplementary material for: Factors affecting nursing and health technician students' satisfaction with distance learning during the COVID-19 pandemic in Morocco: a descriptive study
Source: J Educ Eval Health Prof. 2022 Oct 17;19:28. doi: 10.3352/jeehp.2022.19.28 (PMC9773103; doi:10.3352/jeehp.2022.19.28)
Supplement: Supplementary file 2 — Supplement 1. Survey questionnaires on satisfaction with distance learning during the COVID-19 pandemic in Morocco. [file jeehp-19-28-suppl.docx]

| You are invited to participate in this research project to analyze and evaluate health professions students' satisfaction with distance learning during the Covid-19 pandemic. The data collected during this project is confidential and your anonymity is guaranteed when the results are published. |
| --- |
| I declare my consent to participate in this research:  /__/ Yes /__/ No > |

| How old are you? | /__/__/ years old |
| --- | --- |
| What is your gender? | /__/ Male /__/ Female |
| What is your nationality? | /__/ Moroccan /__/ Others to be specified ……………………………… |
| Which discipline did you follow in your formation? | /__/ Polyvalent Nurse  /__/ Midwife  /__/ Nurse in anesthesia and intensive care  /__/ Emergency and Critical Care Nurse  /__/ Radiology Technician  /__/ Laboratory Technician |
| What is your study level? | /__/ 1st year /__/ 2nd year /__/ 3rd year |
| The platform/application you use mostly in distance learning courses is: | /__/ Google classroom /__/ Moodle /__/ Zoom Cloud meeting /__/ Google Meet /__/ Edmodoo /__/ WhatsApp /__/ Others to be specified: ……………………………………… |
| The internet quality during the distance teaching is: | /__/ Excellent /__/ Good /__/ Poor |

| **The statements below are related to your distance learning courses. Answer these statements by choosing the number that corresponds best to your level of agreement (1= Strongly disagree, 2= Disagree, 3= Neither agree nor disagree, 4= Agree, 5 = Strongly agree)** | | | | | |
| --- | --- | --- | --- | --- | --- |
|  | **1** | **2** | **3** | **4** | **5** |
| There was clear communication of class assignments | **☐** | **☐** | **☐** | **☐** | **☐** |
| Evaluation, test and feedback were given on time. | **☐** | **☐** | **☐** | **☐** | **☐** |
| I felt a part of the class and belonged to the online session. | **☐** | **☐** | **☐** | **☐** | **☐** |
| I am satisfied with faculty accessibility and availability. | **☐** | **☐** | **☐** | **☐** | **☐** |
| I am satisfied with online discussion forums | **☐** | **☐** | **☐** | **☐** | **☐** |
| I am satisfied with online communication including email and announcements. | **☐** | **☐** | **☐** | **☐** | **☐** |
| The platform/application is user-friendly. | **☐** | **☐** | **☐** | **☐** | **☐** |
| I am satisfied with the download duration of learning resources. | **☐** | **☐** | **☐** | **☐** | **☐** |
| I am satisfied with the number of online sessions. | **☐** | **☐** | **☐** | **☐** | **☐** |
| Online courses offered flexible timing. | **☐** | **☐** | **☐** | **☐** | **☐** |
| I am satisfied with the self-directed responsibilities assigned to me. | **☐** | **☐** | **☐** | **☐** | **☐** |
| I enjoyed working on projects during online learning. | **☐** | **☐** | **☐** | **☐** | **☐** |
| I am satisfied with the quality of interaction between me, the faculty and peers. | **☐** | **☐** | **☐** | **☐** | **☐** |
| I am satisfied with collaborative activities during online learning. | **☐** | **☐** | **☐** | **☐** | **☐** |
| I can relate my level of understanding to other students’. | **☐** | **☐** | **☐** | **☐** | **☐** |
| I am comfortable with participating in online sessions | **☐** | **☐** | **☐** | **☐** | **☐** |
| I am satisfied with the level of required effort in online course. | **☐** | **☐** | **☐** | **☐** | **☐** |
| I am satisfied with my performance in online course | **☐** | **☐** | **☐** | **☐** | **☐** |
| I will be satisfied with my final grade | **☐** | **☐** | **☐** | **☐** | **☐** |
| I am able to apply what I learned in this online course. | **☐** | **☐** | **☐** | **☐** | **☐** |
| I will recommend this online learning experience to others. | **☐** | **☐** | **☐** | **☐** | **☐** |
| I am more satisfied with online learning compared to face-to-face sessions | **☐** | **☐** | **☐** | **☐** | **☐** |
| My satisfaction level encourages me to register in other available online | **☐** | **☐** | **☐** | **☐** | **☐** |
| Overall, I am satisfied with this course. | **☐** | **☐** | **☐** | **☐** | **☐** |

***Thank you for your participation***
